# Machine learning based on computational fluid dynamics enables geometric design optimisation of the NeoVAD blades

**DOI:** 10.1038/s41598-023-33708-9

**Published:** 2023-05-03

**Authors:** Lee Nissim, Shweta Karnik, P. Alex Smith, Yaxin Wang, O. Howard Frazier, Katharine H. Fraser

**Affiliations:** 1 Department of Mechanical Engineering, University of Bath, Bath, BA2 7AY UK; 2 Innovative Device and Engineering Applications (IDEA) Lab, Texas Heart Institute, Houston, Texas 77030 USA; 3 Centre for Therapeutic Innovation, University of Bath, Bath, BA2 7AY UK

**Keywords:** Cardiac device therapy, Fluid dynamics, Biomedical engineering, Mechanical engineering

## Abstract

The NeoVAD is a proposed paediatric axial-flow Left Ventricular Assist Device (LVAD), small enough to be implanted in infants. The design of the impeller and diffuser blades is important for hydrodynamic performance and haemocompatibility of the pump. This study aimed to optimise the blades for pump efficiency using Computational Fluid Dynamics (CFD), machine learning and global optimisation. Meshing of each design typically included 6 million hexahedral elements and a Shear Stress Transport turbulence model was used to close the Reynolds Averaged Navier–Stokes equations. CFD models of 32 base geometries, operating at 8 flow rates between 0.5 and 4 L/min, were created to match experimental studies. These were validated by comparison of the pressure-flow and efficiency-flow curves with those experimentally measured for all base prototype pumps. A surrogate model was required to allow the optimisation routine to conduct an efficient search; a multi-linear regression, Gaussian Process Regression and a Bayesian Regularised Artificial Neural Network predicted the optimisation objective at design points not explicitly simulated. A Genetic Algorithm was used to search for an optimal design. The optimised design offered a 5.51% increase in efficiency at design point (a 20.9% performance increase) as compared to the best performing pump from the 32 base designs. An optimisation method for the blade design of LVADs has been shown to work for a single objective function and future work will consider multi-objective optimisation.

## Introduction

Instances of paediatric heart failure due to congenital heart disease (CHD) are between 1 and 2 cases per 1000 births. These patients are in need of heart transplants, however the number of donor hearts available are insufficient to meet this need^1–3^. Left Ventricular Assist Devices (LVADs), can keep patients alive while awaiting a new heart—a so called bridge-to-transplant therapy.

The current LVAD options for paediatric patients all have major limitations. The Berlin Heart EXCOR is an extracorporeal, pneumatically driven, pulsatile flow VAD; although designed specifically for paediatric patients it still has a 20–30% risk of neurological complications due mainly to thrombus formation on the valves^4–6^. The PediMag is an extracorporeal, magnetically levitated, centrifugal VAD, which is only approved for use up to 6 hours and is associated with both infections and neurological events^7–9^. An alternative current solution is to repurpose an existing LVAD designed for adults: the HeartMate II and HVAD have both been used this way, and in 2020 the HeartMate 3 was approved for paediatric patients and is now the most frequently used^6,10,11^. As these pumps were designed for adults with larger cardiac outputs, any operation at the lower flow-rate and pressure-head required by paediatric patients involves reducing the operating speed such that the device is operating off-design so leading to increased blood residence time, blood stasis and thrombosis^1,3,12^. Due to the size of the device the HeartMate 3 has only been implanted in children with a body surface area over 0.78 m$^2$ (19.1 kg), compared to a minimum BSA around 0.6 m$^2$ (13.1 kg) for the HVAD^11,13^. Size limitations mean that patients smaller than this are required to have an extracorporeally located device which is always a risk for infection.

The NeoVAD is a proposed paediatric Left Ventricular Assist Device, small enough to be implanted in infants between 5 and 20 kg. Patients smaller than 5 kg pose a greater challenge for LVAD support generally and the use of continuous-flow devices for these patients has not been well studied^3^. With the added difficulty of being fully implantable, the NeoVAD is not being designed specifically for patients smaller than this limit. The need for a fully implantable LVAD, designed specifically for paediatric patients is urgent and the aim of the NeoVAD is to provide a safe, long-term, bridge-to-transplant therapy that meets this specific need.

The design of the LVAD blade geometry is often incremental and comparative, with the design engineer using experience and sensitivity studies to guide the design^14–18^. Algorithmic optimisation of LVADs is a much less explored design method, however it has been implemented successfully on occasion^19–21^. Optimisation algorithms function by frequently evaluating the underlying function to be optimised and for complex fluid problems such as the design analysis of LVAD blade geometry, this underlying function involves extensive computational fluid dynamics (CFD) simulations. Zhu et. al. used algorithmic optimisation to aid the design of an axial diffuser with the objective of maximising pressure head and minimising backflow in the impeller-diffuser connecting region^19^ showing that even a modest 20 generations of a Genetic Algorithm routine required 1637 CFD simulations and took 37 continuous days to run. More recently, surrogate modelling techniques have been used to minimise the computational expense of design optimisation with notable studies including those by Ghadmi et al. who used an Artificial Neural Network approach to reduce the number of CFD simulations to 400 when optimising the blade shapes of a radial-flow LVAD^20,21^. Of note, although outside the remit of mechanical circulatory support, a study by Grechy et. al. used a Kriging method (a Machine Learning method using Gaussian Process Regression) to produce a surrogate model with only 91 required simulations that led to the improved geometry of arterio-venous fistulae for suppressing unsteady flow^22^.

Building on the works of Ghadmi et. al. and Grechy et. al.^20–22^, this study aims to use a machine learning enabled surrogate model based on CFD simulations to optimise the blade designs of both the impeller and diffuser of the NeoVAD. The objective of maximising efficiency has been chosen to allow for the smallest possible motor to power the device. As the device is designed to be fully implantable, size reduction of the motor is of great concern and as such work is also being undertaken to optimise the design of the blood-contacting motors and their impact on haemolysis^23^. Maximising efficiency also ensures that dissipated energy is minimised and there are proposed links between dissipated energy and blood damage^24^.

## Methods

**Figure 1:**
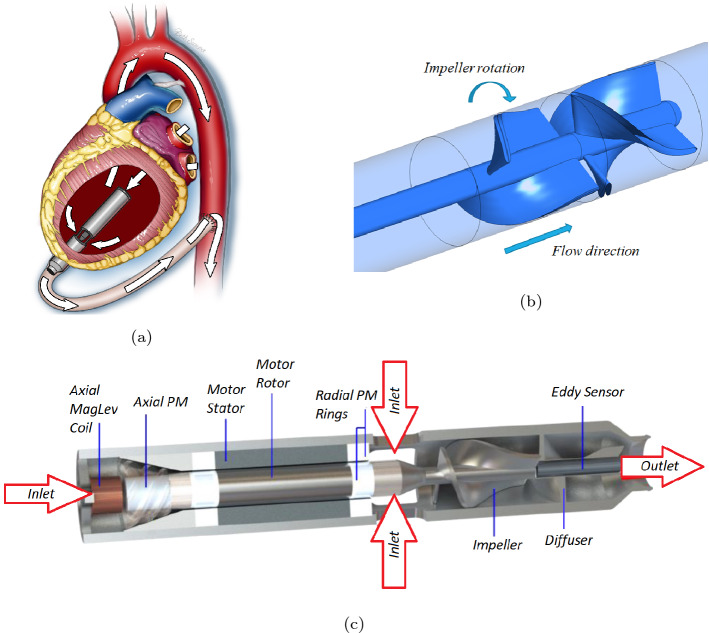
(**a**) Implantation schematic of the NeoVAD in the left ventricle, (**b**) blade configuration inside the NeoVAD (image created in Ansys academic research CFX, release 21.1, https://www.ansys.com/products/fluids/ansys-cfx) (**c**) location of the blades inside the NeoVAD and other features including the MagLev motor and permanent magnets (PM) (image created in Dassault systèmes SOLIDWORKS 2020 https://www.3ds.com/products-services/solidworks/).

Figure 1a shows the proposed implant method for the NeoVAD, and although the specific speed, $N_s = 1.762$ (operating at 15,000 rpm), of the proposed pump lies within the mixed-flow regime of a Cordier diagram^25,26^, size constraints limit the design to being an axial-flow device. The intended paediatric population is 5–20 kg. The target operating point for the smallest babies is a pressure head of 50 mmHg and flow rate 0.5 L/min while at the upper end of the range the target is 70 mmHg and 2 L/min. The initial target operating condition was the upper end of the scale and subsequently optimisation for the middle and low end of the scale was investigated.

A typical blade setup for the NeoVAD is shown in Fig. 1b. Within this study, the naming convention is such that the rotating blade section is referred to as the *impeller* and the stationary section in referred to as the *diffuser*. The *rotor-stator* convention that is more common for axial-flow devices is avoided to reduce confusion with the magnetic levitation and motor parts that bear the same name. As can be seen from Fig. 1b the impeller consists of 2 rotating blades and the diffuser consists of 3 stationary blades, in keeping with the previous study by Smith et al.^27^. A cutaway view of the NeoVAD is shown in Fig. 1c. This view shows an overview of the NeoVAD highlighting the location of the impeller and diffuser blades, the motor rotor and motor stator, maglev bearing and associated permanent magnets, and inlets and outlet of blood flow.

### Computational fluid dynamics

The blade geometry for this study is defined and parameterised in accordance with previous experiments^27^. The baseline design consists of an impeller of two blades and a diffuser of three blades. The blades themselves are of circular arc design and have a constant thickness of 0.5 mm and elliptical leading and trailing edges. Only the free parameters defined in previous experiments^27^ were considered for this study, such that each design simulation had an experimental counterpart, and to avoid introducing additional free parameters that could increase computational expense.

**Figure 2:**
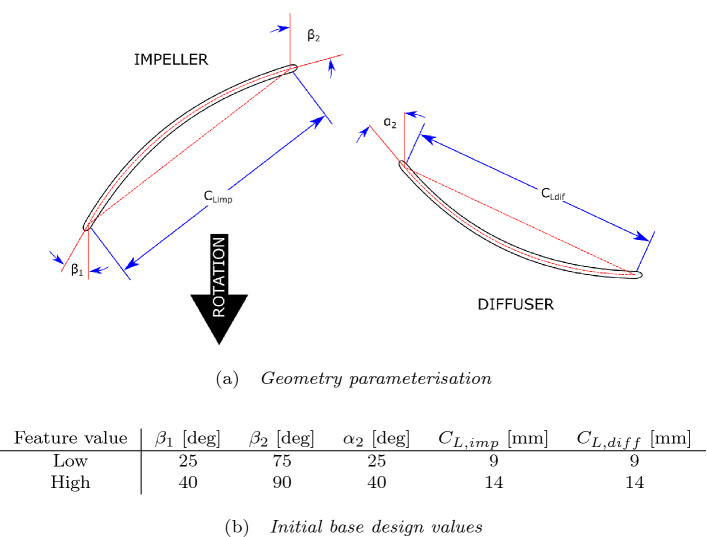
Parameterisation of the mid-span blade shapes into five governing parameters: impeller inlet angle, $\beta _1$, impeller outlet angle, $\beta _2$, diffuser inlet angle, $\alpha _2$, impeller chord length, $C_{L,imp}$ and diffuser chord length, $C_{L,diff}$. Figure adapted from Smith et al.^27^.

Five variable parameters govern the blade shape as can be seen in Fig. 2a, namely the inlet angles and chord lengths of both impeller and diffuser blades and also the outlet angle of the impeller. The outlet angle of the diffuser is set to 90 degrees (as measured from the tangential direction) so as to align the downstream flow axially. Using this method, the camber line at mid-span can be calculated for any specified values of these five parameters.

*Ansys*®*Academic Research BladeGen, Release 21.1 (Ansys Inc., Canonsburg, Pennsylvania, United States)* was used to create the full impeller diffuser geometry by sweeping the profile between the hub, with a radius of 1 mm, to the shroud, with a radius of 3.85 mm. The full geometry was exported from *BladeGen* to *Ansys*®*Academic Research TurboGrid, Release 21.1 (Ansys Inc.)* where an impeller tip gap of 100 $\mu$m was added and a hexahedral mesh was created. The process, from specification of input parameters through to mesh creation was automated and controlled using a script created in *MATLAB*®*R2020b (The MathWorks, Inc., Natick, Massachusetts, United States)*.

Smith et. al.^27^ used the parameterisation method shown here to create and test 32 different pump designs that consisted of 8 unique impellers and 4 unique diffusers. To do this, each of the 5 geometry-governing parameters was assigned a *high* and a *low* value, which can be seen in Fig. 2b. Combining these values in every configuration gives the 32 base designs.

Computational fluid dynamics simulation utilised *Ansys*®*Academic Research CFX, Release 21.1 (Ansys Inc.)* to solve either the Reynolds Averaged Navier Stokes (RANS) or unsteady RANS using a shear stress transport turbulence model, the SST k-$\omega$. The flow field experiences a range of Reynolds numbers, with pipe flow based estimates in the inlet region spanning $\text {Re} = {vD\rho }/{\mu } = 100 - 850$, where *v* is the average velocity based on the flow rate span of $Q = 0.5 - 4$ L/min, *D* the diameter (7.7 mm), $\rho$ the density (taken as 1050 kg/m$^3$^28^) and $\mu$ the viscosity (taken as 0.0035 Pa$\cdot$s^28^). Using the rotor tip velocity for estimates in the rotating section, the Reynolds number can reach $\text {Re} = {\omega D^2 \rho }/{\mu } = 37,000$, using an estimated maximum rotating speed, $\omega = 20,000$ rpm. The total range of Reynolds number in the flow field puts this device in the transitional regime. The SST k-$\omega$ model has been widely used in previous studies of rotary mechanical circulatory support devices^28–30^.

Fourth-order Rhie Chow pressure-velocity coupling was used and a blended spatial discretisation scheme that utilises second-order central differencing but using a blending function to introduce enough first-order upwind differencing to prevent overshoots when the local solution gradient is large^31^.

#### Mesh dependence

**Figure 3:**
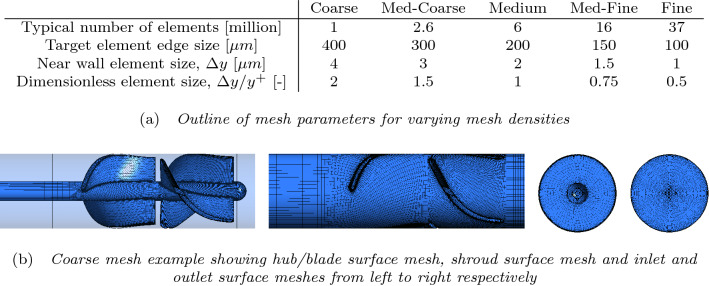
Comparison of mesh creation parameters for mesh density study and an example mesh illustrating hexahedral element structure and expansion ratios.

To explore the effect of the mesh density on the solution, a pump design was chosen at random from a list of 32 geometries that were studied experimentally. The default mesh density was set such that the first near wall elements were sufficiently small for a target of $y^{+} < 1$. Other density meshes were created both coarser and finer such that solution comparisons could be made. Mesh parameters can be seen in Fig. 3a. Away from the wall, mesh element lengths were expanded smoothly and an example of the coarsest mesh can be seen in Fig. 3b.

Mesh dependency was evaluated for steady-state simulations utilising the mixing plane boundary method between the rotating and stationary frames of reference, which averages the pressure and velocity fields circumferentially at the reference frame boundary. Transient Unsteady RANS (URANS) and Detached Eddy Simulation (DES) simulations were also run for comparison, using the sliding plane boundary method, which conserves the pressure and velocity field across the boundary. In all instances the boundary is located at the midpoint between the impeller trailing edge and the diffuser leading edge. The transient simulations have a time-step length equivalent to $5^\circ$ of rotation per time-step and run for 50 full rotations of the pump and the last 20 full rotations are averaged to find the pressure and efficiency results. Transient simulations are inherently more computationally expensive, and the mesh study allowed comparison of different density meshes but also a comparison of temporal solution methods.

#### Scaling

With designs that have been experimentally tested, the rotational speed required to pass through the given operating point of $Q = 2$ L/min, $H = 70$ mmHg has already been found. For designs to be simulated without prior knowledge of the required rotation speed, dimensional analysis was used. Simulations were carried out at an estimated speed (given that this estimate does not take the pump into an entirely different turbulence regime) and the resulting pressure-flow (*HQ*) and efficiency-flow ($\eta Q$) curves were scaled such that they fit the desired operating point. This gave the required rotating speed, that differed from the original estimate.

The 32 pump designs were simulated with a rotating speed of 20,000 rpm in the first instance. To scale the *HQ* curves such that they passed through the required operating point, a quartic polynomial was fit to the data, with the form

1 $$\begin{aligned} \Delta P = c_1 Q^4 + c_2 Q^3 + c_3 Q^2 + c_4 Q + c_5 \end{aligned}$$

The coefficients of this equation were found using *MATLAB* curve fitting tools.

Using the definition of the flow and pressure coefficients

2 $$\begin{aligned} \phi = \frac{Q}{\omega D^3}, \quad \psi =\frac{\Delta P}{\rho \omega ^2 D^2} \end{aligned}$$

it is possible to arrive at the similitude scaling laws between two operating points, which can be arranged such that

3 $$\begin{aligned} \Delta P_2 = \Delta P_1 \left( \frac{Q_2}{Q_1}\right) ^2 \end{aligned}$$

where the subscript 1 describes the desired operating point and subscript 2 describes the operating point on the resultant *HQ* curve at 20,000 rpm that shares the dimensionless coefficients, $\phi$ and $\psi$.

As the exact point on the resultant *HQ* curve that shares these dimensionless coefficients is unknown, we can substitute the quartic polynomial that describes the entire curve knowing that

4 $$\begin{aligned} \Delta P_2 = c_1 Q_2^4 + c_2 Q_2^3 + c_3 Q_2^2 + c_4 Q_2 + c_5. \end{aligned}$$

Equating Eqs. (3) and (4) and rearranging gives

5 $$\begin{aligned} c_1 Q_2^4 + c_2 Q_2^3 + \left( c_3 - \frac{\Delta P_1}{Q_1^2} \right) Q_2^2 + c_4 Q_2 + c_5 = 0 \end{aligned}$$

With the coefficients, $c_{1 ... 5}$ known, solving for $Q_2$ allowed the calculation of $P_2$ from the resultant *HQ* curve and subsequently the rotating speed $\omega _1$ that resulted in the curve passing the desired operating point. Using the dimensionless coefficients of Eq. (2), the *HQ* curve and efficiency curve were rescaled.

Using this method, *HQ* and $\eta Q$ curves can also be scaled to other operating points, namely $OP_{1\textrm{L}/\textrm{min}}: \Delta P = 60\,\text {mmHg}, Q = 1\,\text {L/min}$ and $OP_{0.5\textrm{L}/\textrm{min}}: \Delta P = 50\,\text {mmHg}, Q = 0.5\,\text {L/min}$. This allows the final optimised design—designed to maximise efficiency at $OP_{2\textrm{L}/\textrm{min}}: \Delta P = 70\,\text {mmHg}, Q = 2\,\text {L/min}$—to be evaluated for performance across a range of conditions expected throughout the development of a paediatric patient.

### Surrogate model

Commonly implemented optimisation routines operate by regularly evaluating the underlying function. In the case of this study—optimising geometry for maximum efficiency—each function call would involve specifying the values of the five governing parameters, creating the geometry and mesh and running CFD simulations at multiple flow rates such that pressure- and efficiency-flow curves can be created. The former for scaling the results to the desired operating point and the latter for identifying the efficiency at the given design point of $\Delta P =70$ mmHg, $Q = 2$ L/min. This approach is far too computationally expensive and therefore it is desirable to use the CFD simulation results that we already have to create a surrogate model that can predict efficiency values at geometry designs not previously simulated. There are many methods for the creation of surrogate models^32^, two machine learning methods, namely Gaussian Process Regression and Artificial Neural Networks, were used to create surrogate models and these were compared with a simple multi-linear regression model benchmark, created using least squares regression in *MATLAB*.

Gaussian Process Regression—often called simple Kriging—is a machine learning tool well suited for small problems. A Gaussian Process Regression model was implemented in *MATLAB* using a five-fold cross-validation method whereby the data is partitioned to exclude a fifth of available set for training a validation. This occurs five times and an average fit quality is calculated and becomes the target for a model trained on all available data. This method protects against over-fitting whilst using all available data, a great benefit for small data-sets like the set in this study^33^. A range of different kernel models were implemented (Exponential, Squared Exponential, Matern 5/2 and Rational Quadratic), of which the Matern 5/2 method was chosen as it resulted in the best predictive ability.

Neural networks are generally best suited for large data-sets and are regularly implemented on sets with over a million results. To discern whether neural networks could be useful in creating a surrogate model for optimisation in this instance, a Bayesian Regularised Artificial Neural Network (BRANN) was trained in *MATLAB*. The benefit of using Bayesian regularisation is that it eliminated the possibility of over-fitting and allowed a holdout validation to be carried out with just a training and validation set, rather than a training, testing and validation set as would be required to protect from over-fitting in a non-regularised Artificial Neural Network training method^34^. The BRANN in this study used a holdout set of 5 of the 32 designs (15%) and was created with a single hidden layer of five neurons as including more neurons failed to improve the model. Input scaling was performed such that each geometry parameter and target efficiencies ranged from $-$ 1 to 1.

The objective that the surrogate models are tasked to predict is the efficiency, $\eta$, at the design point of $\Delta P = 70$ mmHg, $Q = 2$ L/min. The inputs to the surrogate consist only of the five geometry-governing parameters, illustrated in Fig. 2. As the pressure- and efficiency-flow curves have been scaled to ensure that the pump is operating at this design point, the efficiency value for each pump design can be linearly interpolated. By specifying the design point in this way, rotating speed becomes intrinsic to the design geometry and does not need to be an input into the surrogate model. Furthermore, specifying one design point also removes the need for the surrogate to predict the entire *HQ* curve at one or multiple rotating speeds and limits the target values to a single objective per design. Overall, the surrogate has 5 inputs—the geometric parameters—and 1 output—the efficiency at $\Delta P = 70$ mmHg, $Q = 2$ L/min. To produce full *HQ* and $\eta Q$ curves for the resulting optimised design, a series of simulations is required. These simulations are carried out at 20,000 rpm and scaled to the design operating point, which reveals the required rotating speed for this geometry.

### Optimisation

A genetic algorithm was used to optimise the geometric design of the mid-span blade shapes, using each of the three surrogate models as the underlying function estimating efficiency. More precisely, the optimisation routine acts to minimise the negated value of efficiency at the operating point $\Delta P = 70$ mmHg, $Q = 2$ L/min.

The Genetic Algorithm was implemented in *MATLAB* and used a Gaussian mutation method with a crossover fraction of 0.8 and a population size of 50. Convergence was reached when the relative change in the generation’s best function evaluation (i.e. negative of efficiency) was less than or equal to $10^{-6}$. The input parameters were subject to the constraint $\beta _2 > \beta _1$ such that the impeller blade was correctly oriented and moreover each parameter was also subjected to boundary constraints which limited the range of values.

The purpose of the boundary constraints are to examine the ability of the surrogate models and the optimisation routine to search and find designs that may lay outside the neighbourhood of the original training data. For this investigation, an iterative approach to the boundary constraints has been implemented.

**Table 1:** Sequential boundary constraint iterations for the optimisation of the five geometry-governing parameters, showing minimum and maximum values permitted for each parameter.

Constraint iteration	$\beta _1$ [deg]		$\beta _2$ [deg]		$\alpha _2$ [deg]		$C_{L,imp}$ [mm]		$C_{L,diff}$ [mm]	
	min.	max.	min.	max.	min.	max.	min.	max.	min.	max.
0	25	40	75	90	25	40	9	14	9	14
1	20	45	70	90	20	45	8	15	8	15
2	15	50	65	90	15	50	7	16	7	16
3	10	55	60	90	10	55	6	17	6	17
4	5	60	55	90	5	60	5	18	5	18

Table 1 shows the allowable ranges for the five input parameters over four iterations, named *Constraint Iteration 0 ... 3*. It can be seen that *Constraint Iteration 0* uses the extremes of the original 32 simulations as the range boundaries, thus allowing the optimisation routine to use the parameter space that represents purely interpolation between existing designs. As the iterations continue, the ranges get wider and allow the searchable parameter space to include regions where the surrogate models are extrapolating but which may contain more efficient designs.

As there are no extra simulations to run, it is straightforward to create new surrogate models at different operating conditions based on the rescaled data from the 32 pump design simulation results. This then allowed the optimisation of pump designs that maximise efficiency at $OP_{1\textrm{L}/\textrm{min}}$ and $OP_{0.5\textrm{L}/\textrm{min}}$ for comparison with the optimised design at $OP_{2\textrm{L}/\textrm{min}}$.

## Results

### Computational fluid dynamics

**Figure 4:**
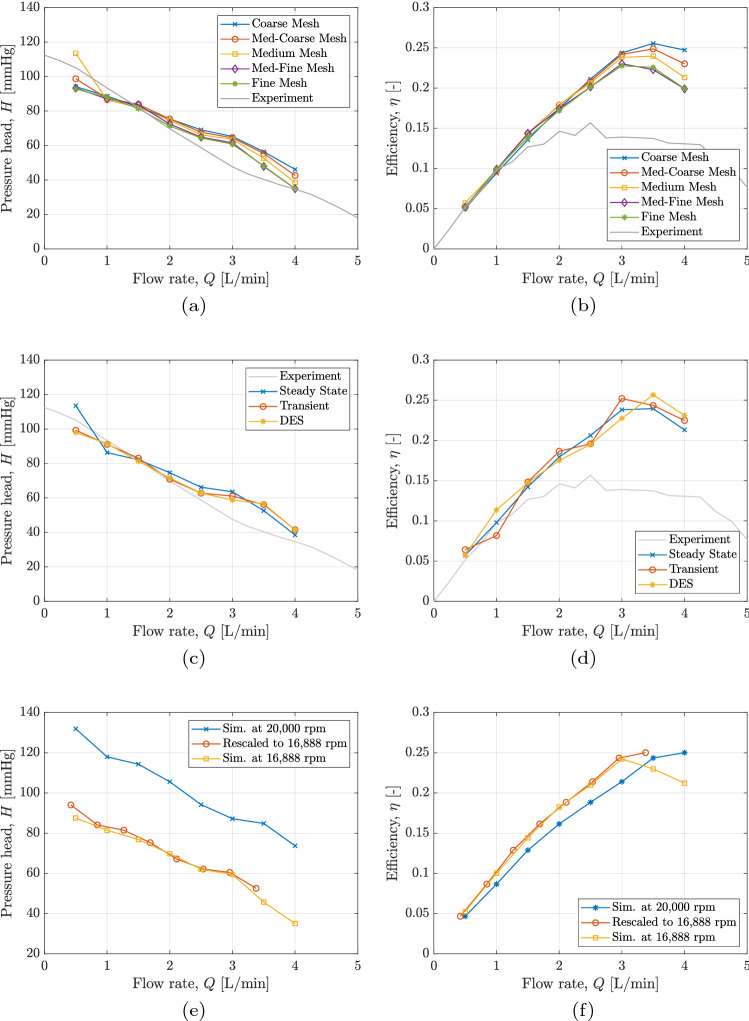
(**a**) Pressure-Flow curves and (**b**) Efficiency-Flow curves for steady state mesh density tests run at $\omega = 17,350$ rpm to match experiments. (**c**) Medium density mesh comparison of steady-state simulation using the mixing plane boundary method for rotating frames of reference and full transient URANS and DES simulations using the sliding plane boundary method showing the resulting *HQ* curves and (**d**) shows the resulting $\eta Q$ curves. Plots (**e**) and (**f**) show the results of the scaling procedure on simulations at 20,000 rpm compared to simulations carried out explicitly at the operating point found to satisfy the operating condition.

The steady-state simulation results for the randomly selected pump design show that although there are discrepancies between the simulation results and the experimental results, the greatest deviations are seen at higher flow rates. The results of these simulations can be seen in Fig. 4a,b. In the region of interest (0.5–2 L), the simulations agree well with an RMSE value of 11.07 mmHg between the coarsest mesh and the experimental results, and 7.50 mmHg between the finest mesh and the experimental results. It can be seen that differences between solutions over different mesh densities are also greatest at larger flow rates whereas in the area of interest, the comparison of mesh densities shows only small deviations between solutions on different meshes with RMSE values of 5.64 mmHg for the *coarse*, 4.92 mmHg for the *medium-coarse*, 2.88 mmHg for the *medium* (excluding the anomalous value at 0.5 L/min) and 1.00 mmHg for the *medium-fine* meshes when compared to the *fine* mesh. The mesh density termed *medium* was selected going forward, with typically 6 million hexahedral elements.

The steady-state simulations conducted struggle to converge to the desired $10^{-4}$ threshold for RMS momentum residuals. With the exception of the densest mesh tested, all mesh simulations reach a momentum residual level in the range 3–6 $\times 10^{-4}$ and reduce no further. The values of interest, namely the pressure head and efficiency, steadily oscillate around a fixed point, and by averaging the last 200 iterations of a 1000 iteration simulation, this point can be found. This is not unexpected for a flow field that is inherently transient in nature and when a fully transient simulation is run this issue disappears.

Figure 4c,d shows a comparison between a steady-state simulation (using the mixing plane boundary method between the rotating and stationary frames of reference) and both transient URANS and DES simulations (using the sliding plane boundary). For the case of pressure head and efficiency, the difference between the *’pseudo-converged’* steady-state results and both fully converged transient results are minimal, with RMSE between RANS and URANS being 5.89 mmHg, between RANS and DES being 6.47 mmHg and between URANS and DES being 1.19 mmHg. The benefit of reduced computational expense in steady-state simulations cannot be ignored, and therefore steady-state simulations have been deemed sufficient for this study. Steady-state simulations on the *medium* mesh took on average 240 CPU hours on Microsoft Azure HBv3 Virtual Machines featuring AMD EPYC™7V73X (Milan-X) CPU cores. Transient simulations on the same mesh using the same CPU cores took 688 CPU hours.

The deviation of simulation results from experimental results at larger flow rates is present to the same degree across RANS, URANS, and DES simulations, which suggests that this inaccuracy is independent of the transient nature and of the turbulence model. It is believed that this inaccuracy is due to the surface roughness of the 3D printed testing prototypes, which at length scales required for paediatric pumps may play a large effect, especially on efficiency. Further study into the effect of surface roughness and the modelling of this effect is underway.

To validate the applicability of the scaling method, the 32 base pump designs were re-simulated at their respective found rotating speeds and these simulation results compared to the original curves. An example of this method can be seen in Fig. 4e,f, whereby the randomly selected test pump has been simulated at a rotating speed of 20,000 rpm, scaled such that the curve passes through the required operating point of $\Delta P = 70$ mmHg, $Q = 2$ L/min, resulting in a scaled operating speed of 16,888 rpm, and finally a series of simulations at this rotating speed. The root mean squared error (RMSE) between the scaled curve and the explicitly simulated 16,888 rpm HQ curves is 2.09 mmHg. Across all 32 pump designs, RMSE was calculated between scaled *HQ* curves and re-simulated *HQ* curves and the average RMSE was just 3.14 mmHg. The average RMSE between scaled *HQ* curves and experimental results was 8.81 mmHg and the calculated rotating speeds, $\omega$, from this method differed to the values attained experimentally on average by only 4.3%. This method was therefore deemed appropriate for scaling simulation results to the design operating point.

### Surrogate model

**Figure 5:**
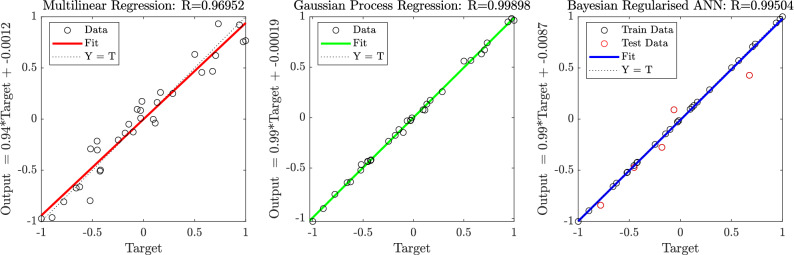
Regression analysis of baseline Multilinear Regression and Machine Learning methods—Gaussian Process Regression and Bayesian Regularised Artificial Neural Network. *Output* along the *y*-axis shows the normalised output of the surrogate at each of the 32 designs and *Target* along the *x*-axis shows normalised simulations results for efficiency at these design points.

To compare the performance of each of the surrogate modelling methods, RMSE values were compared. In the case of the multi-linear regression, which involved a simple least squares regression method, there is no specific validation or testing set, and as such the model is trained on all available data. The resulting RMSE of model predicted points across all 32 designs compared to the target values from simulations of all 32 designs was 0.0104 (This value is the dimensionless measure of efficiency and as such could be read as 1.04%). The normalised predictions of efficiency resulting from this model as compared to the simulations can be seen in Fig. 5 alongside those of the Gaussian Process Regression (GPR) and the Bayesian Regularised Artificial Neural Network (BRANN). Values for the coefficients of the least-squares multi-linear regression can be found in the Supplementary Material in Eq. S1.

In the case of the GPR, 5-fold cross-validation was utilised, the RMSE value for validation which is a measure of the error while each design was being used for testing and not training during each fold was 0.009541. When the predictions of the final model, trained on all data, were compared to simulations—which can be seen in Fig. 5—the RMSE value was 0.001936.

Finally, in the case for the BRANN, holdout validation was utilised and as such there was a specific test set that remained unseen during training, the RMSE value between model predictions and simulations for this test set was 0.01079, for the training set the RMSE was $5.760 \times 10^{-9}$, and across all 32 designs the RMSE was 0.004264. The training set and testing set have been highlighted in Fig. 5.

### Optimisation

The Genetic Algorithm routines at each constraint iteration and acting on each of the surrogate models typically converged after a number of generations in the region of 100 - 130. For an optimisation routine with 130 generations, each of a population of 50 designs, the cost of simulating eight operating points to populate a pressure-flow curve for scaling is 12 million CPU hours (using Microsoft Azure HBv3 Virtual Machines featuring AMD EPYC™7V73X (Milan-X) CPUs). Using a surrogate model, the optimisation routine was able to converge upon a solution never exceeding 2 minutes. For each constraint iteration and surrogate model, the optimisation routine was repeated five times, within which the solution never differed by more than $1\%$ in any parameter.

**Figure 6:**
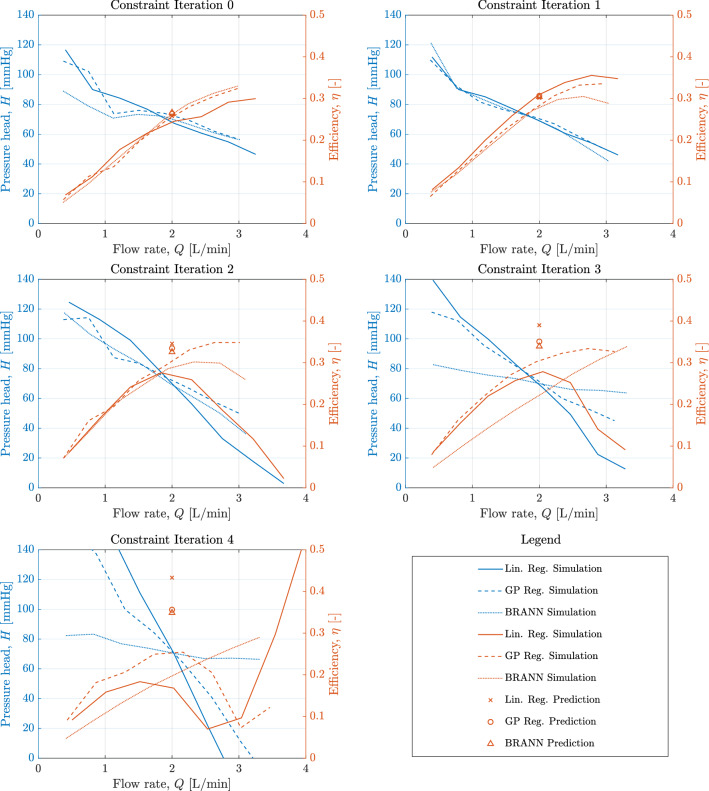
Results of each optimisation routine operating under successive constraint iterations and using each surrogate model as the underlying function evaluation method. Markers show the prediction of efficiency give by each surrogate model at the operating condition, $\Delta P = 70$ mmHg, $Q = 2$ L/min, and the lines show the results of subsequent simulations of the resultant designs.

Figure 6 shows the efficiency-flow curves of each design converged upon by the optimisation routine for each constraint iteration and underlying surrogate model. When operating in or close to the neighbourhood of the simulation designs used to train the surrogate models, the predictions of efficiency at the desired design point align well with the efficiency results of the CFD simulations of these designs. As the model is allowed to extrapolate further from this region, the predictions and resulting simulations deviate from one another. This occurs to differing extents dependant on the surrogate used and it can be seen that the Multi-Linear Regression (MLR) model and the Bayesian Regularised Artificial Neural Network model are less accurate predictors than the Gaussian Process Regression model at both Constraint Iterations 2 and 3. At Constraint Iteration 3, the error in the efficiency predictions for the MLR and BRANN are $47.26 \%$ and $51.36 \%$ respectively, compared with $10.21 \%$ for the GPR. Full details of predicted and simulated efficiencies, errors, and geometry parameters across all iterations are listed in Supplementary Table S1. Moreover the behaviour of the pumps at Iteration 3 suggests that both the multi-linear and BRANN models converge upon pump designs that are operating far from optimally. At Iteration 4, none of the pumps are operating in an optimal way. The GPR informed design at Iteration 3 has the highest efficiency at $Q = 2$ L/min of all the designs simulated.

**Figure 7:**
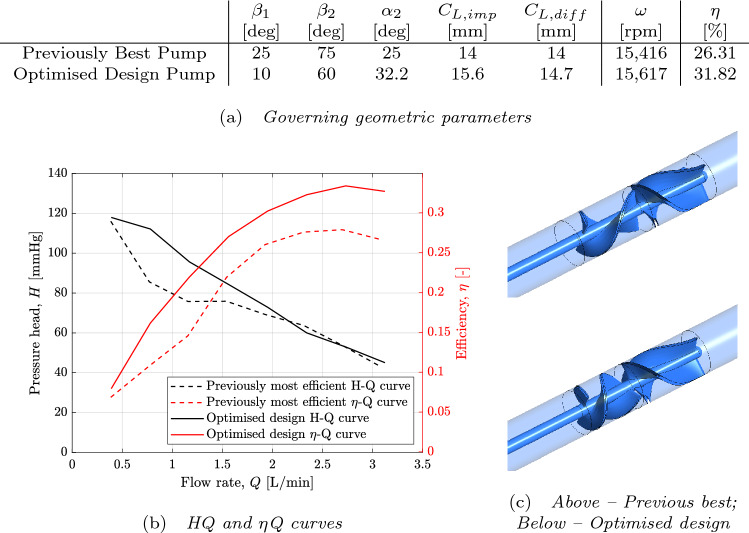
Comparison of previously best performing pump design from the original 32 base designs and the new optimised blade design at selected operating point of Q = 2 L/min, H = 70 mmHg—the result of the optimisation routine utilising constraint iteration 3 and the Gaussian process regression surrogate model.

The governing parameters of this design with the maximum achieved efficiency can be found in Fig. 7a and can be seen alongside the best performing pump of the original 32 designs. The geometry of each of these two designs can be seen in Fig. 7c. It can be seen that even at this constraint iteration, the design of the impeller inlet and outlet angles, $\beta _1$ and $\beta _2$ lie at the extremes of the allowable ranges set out in Table 1. This suggests that the surrogate model believes that greater efficiency can be achieved further outside of these ranges however the accuracy of predictions in this region are poor. Further simulation results in this region of the design space would allow a surrogate model to be trained which could more reliably predict the performance of pump designs in this neighbourhood.

Figure 7b shows the comparison of pump performance between the newly optimised pump design and that of the previously best performing design. The efficiency of the optimised pump at the target design point was $\eta = 0.3182$ and the previously best performing of all 32 base designs was only $\eta = 0.2631$. Although the objective function of the optimisation routine only explicitly maximised efficiency at the target operating point of $\Delta P = 70$ mmHg, $Q = 2$ L/min, the resultant design achieves greater efficiency at all simulated flow rates. A downside of utilising dimensional scaling to drastically reduce computational expense is that simulations are not explicitly run that correspond to the design point in question therefore the flow fields were compared at 20,000 rpm rather than the design point. See Supplementary Material Fig. S1 for a comparison of the flow fields for the previously best performing pump and the newly optimised pump.

Due to the nature of this optimisation methodology, the design was easily studied at other operating conditions, namely $OP_{1\textrm{L}/\textrm{min}}: \Delta P = 60 \text {mmHg}, Q = 1 \text {L/min}$ and $OP_{0.5\textrm{L}/\textrm{min}}: \Delta P = 50 \text {mmHg}, Q = 0.5 \text {L/min}$. By rescaling the *HQ* and $\eta Q$ curves, the optimised design was found to achieve efficiencies of $\eta _{1\textrm{L}/\textrm{min}} = 0.2213$ and $\eta _{0.5\textrm{L}/\textrm{min}} = 0.1528$.

Separate surrogate models, trained on rescaled data from the 32 pump simulations using GPR and Constraint Iteration 3 created new optimised designs specifically for these other design points which resulted in predicted efficiencies of $\eta _{1\textrm{L}/\textrm{min}} =0.2206$ and $\eta _{0.5\textrm{L}/\textrm{min}} = 0.1397$, showing that the original optimised design targeting the operating point at $Q = 2$ L/min, performs as well as specialised designs at these operating points. As there were no extra simulations required for this comparison across operating points, the computational time taken to create these surrogates and optimise the designs is negligible compared to even a single CFD simulation.

## Discussion

The successful implementation of this optimisation method has led to a pump design that operates more efficiently at the design operating condition of $\Delta P = 70$ mmHg, $Q = 2$ L/min, but also at all flow and pressure points along the entire *HQ* curve at the specified rotating speed, allowing assured efficiency increase over the previously best performing pump even off-design. This off-design performance is important for the suitability of the pump for infants that will be growing while having the LVAD implanted.

Issues identified in this study include a discrepancy in simulations and experimental results at higher flow rates—those at 3 L/min and above—above the intended operating points of the NeoVAD. This is believed to be due to the inherent surface roughness of the rapidly prototyped 3D printed experimental models. Further study into this effect is currently being performed.

Another issue identified in the current study is that of extrapolation away from the original neighbourhood of the training simulations. This has limited the optimisation routine’s ability to select unconstrained geometry parameters and as such, designs uncovered in this study, although successful, have much room for improvement. The training simulations were chosen to match experimentally tested designs, but future studies will build on this approach by adding more simulation data over a broader range of the parameter space, with attention paid to those areas local to the suspected global minima. One way to achieve this is to feed the simulated designs of optimised pumps back into a new surrogate model to continually refine the surrogate in the region of the optimal design, while another is to more creatively select the initial base designs with a rigorous design of experiments approach.

Utilising a surrogate model approach is vital for keeping computational expense feasible for a Genetic Algorithm routine and care must be taken over the method of surrogate model creation. Gaining the maximum information from a small available data-set requires the use of more complex regression than simple multi-linear methods and it has been shown that Gaussian Process Regression is a more suitable choice for such limited data-sets than Artificial Neural Network methods, despite Bayesian regularisation. In studies where training data is less computationally expensive to generate—either by advancement of simulation techniques or by simpler problem geometry—BRANN surrogate modelling may become the preferred option.

Maximising efficiency is important for LVADs as a way to minimise the electrical power required through either a driveline or a transcutaneous electrical transmission method. Minimising power requirements is especially important for the NeoVAD as the motor is required to fit inside the patient’s ventricle. The greater the efficiency in turning electrical power into the required fluid power, the smaller the driving motor can become. The hypothesised link between reducing unused dissipative energy and reducing blood damage^24^ also implicates energy as a good optimisation criterion. Although efficiency has been selected as the design objective in this instance, the method will enable the further optimisation of more complex design considerations such as for minimising haemolytic damage or thrombus potential. There are further challenges when expanding the research to cover these objective functions, most notably the use of dimensional analysis methods to scale hydraulic parameters such as efficiency, which are not so easily utilised for blood damage. Developing such scaling laws for haemolysis is an avenue for further research.

Also notable in this study is the potential for change in shape of the pressure-flow *HQ* curve. The increase in efficiency performance comes with a significant rise in pressure head at lower flow-rates. This may well be desirable as it allows for a lower pump rotating speed when operating in this region, however, the gradient of the *HQ* curve is also important for the design of LVADs. Shallow gradients allow for greater flow-rate sensitivity to pressure and allow for passive pulsatile behaviour with changing pressure boundary conditions as would be experienced in-vivo. Caution should be exercised with designs that significantly change this behaviour.

In conclusion, an optimisation method for designing the geometry of NeoVAD blades has been successfully implemented for maximising the single objective of efficiency resulting in a 20.96% increase in performance. Future work will focus of increasing the number of variable parameters to include in the optimisation such as blade thickness, tip gap and blade number while also addressing the issue of poor model extrapolation by increasing the parameter space range of training simulations. This method can be implemented for a variety of objective problems without the need for prohibitive CFD simulations for every optimisation routine function evaluation. The ability of these surrogate models to be trained cheaply allowing optimisation routines to be carried out, paired with dimensional rescaling has allowed for the rapid design of pumps at different operating conditions with no requirement for additional simulations: an improvement beyond the methods previously demonstrated in this field. This allows for an adaptable inverse design workflow which would otherwise be unfeasible as an LVAD design strategy.

## Supplementary Information

Supplementary Information.

## Data Availability

The data that support the findings of this study are available from the corresponding author, K.H.F., upon reasonable request. Data will be made available on https://researchdata.bath.ac.uk shortly.
