# Supplementary material for: Machine learning based on computational fluid dynamics enables geometric design optimisation of the NeoVAD blades
Source: Sci Rep. 2023 May 3;13:7183. doi: 10.1038/s41598-023-33708-9 (PMC10156814; doi:10.1038/s41598-023-33708-9)
Supplement: Supplementary file 1 — Supplementary Information. [file 41598_2023_33708_MOESM1_ESM.pdf]

Machine Learning based on Computational Fluid Dynamics enables geometric design optimisation of the NeoVAD blades:
Supplementary material

Tables

	β_1 [deg]	β_2 [deg]	α_2 [deg]	$C_{L,imp}$ [mm]	$C_{L,diff}$ [mm]	Pred. η [-]	Sim. η [-]	Error [%]
MLR 0	25.00	75.00	25.16	9.00	14.00	0.2578	0.2431	6.0469
MLR 1	20.00	70.00	26.71	8.00	15.00	0.3014	0.3097	2.6800
MLR 2	15.00	65.00	15.00	7.00	16.00	0.3458	0.2748	25.8370
MLR 3	10.00	60.00	10.00	6.00	17.00	0.3898	0.2647	47.2611
MLR 4	5.00	55.00	5.00	5.00	18.00	0.4330	0.1729	150.4338
GPR 0	25.00	75.00	33.00	13.94	14.00	0.2633	0.2553	3.1336
GPR 1	20.00	70.00	32.12	15.00	15.00	0.3063	0.2824	8.4632
GPR 2	15.00	65.01	32.09	15.44	15.31	0.3346	0.3070	8.9902
GPR 3	10.00	60.00	32.20	15.63	14.75	0.3507	0.3182	10.2137
GPR 4	5.03	55.03	32.68	15.77	14.42	0.3562	0.2562	39.0710
BRANN 0	25.00	75.00	35.33	12.89	14.00	0.2662	0.2649	0.4908
BRANN 1	20.00	70.00	25.60	15.00	15.00	0.3038	0.2792	8.8109
BRANN 2	15.00	65.01	23.26	16.00	16.00	0.3256	0.2753	18.2710
BRANN 3	10.00	64.30	19.02	17.00	17.00	0.3392	0.2241	51.3610
BRANN 4	5.01	64.31	13.52	17.97	18.00	0.3488	0.1978	76.3397

Table 1. Geometry parameters, predicted efficiency, simulated efficiency and subsequent prediction error for all optimised designs outputed from Multi-Linear regreion (MLR), Gaussian Process Regression (GPR), and Bayesian Regularised Artificial Neural Network (BRANN) across all Constraint Iterations 0 to 4.

Equations

The coefficients of the least-squares multi-linear regression can offer some insight to the relative importance of each geometry parameter for efficiency of the overall design. The multi-linear regression results in the following equation:

$$\eta_{\text{predicted}} = 0.5724 - 0.0039\beta_1 - 0.0034\beta_2 - 0.0001\alpha_2 - 0.0025C_{L,imp} + 0.0046C_{L,diff} \quad (1)$$

Limited information can be drawn from this outside of the range of training data as the multi-linear model performed poorly when extrapolating. A more in-depth statistical analysis of the NeoVAD geometry parameters can be seen in *Smith et. al., Design Method Using Statistical Models for Miniature Left Ventricular Assist Device Hydraulics, Annals of Biomedical Engineering, 2019*

Figures

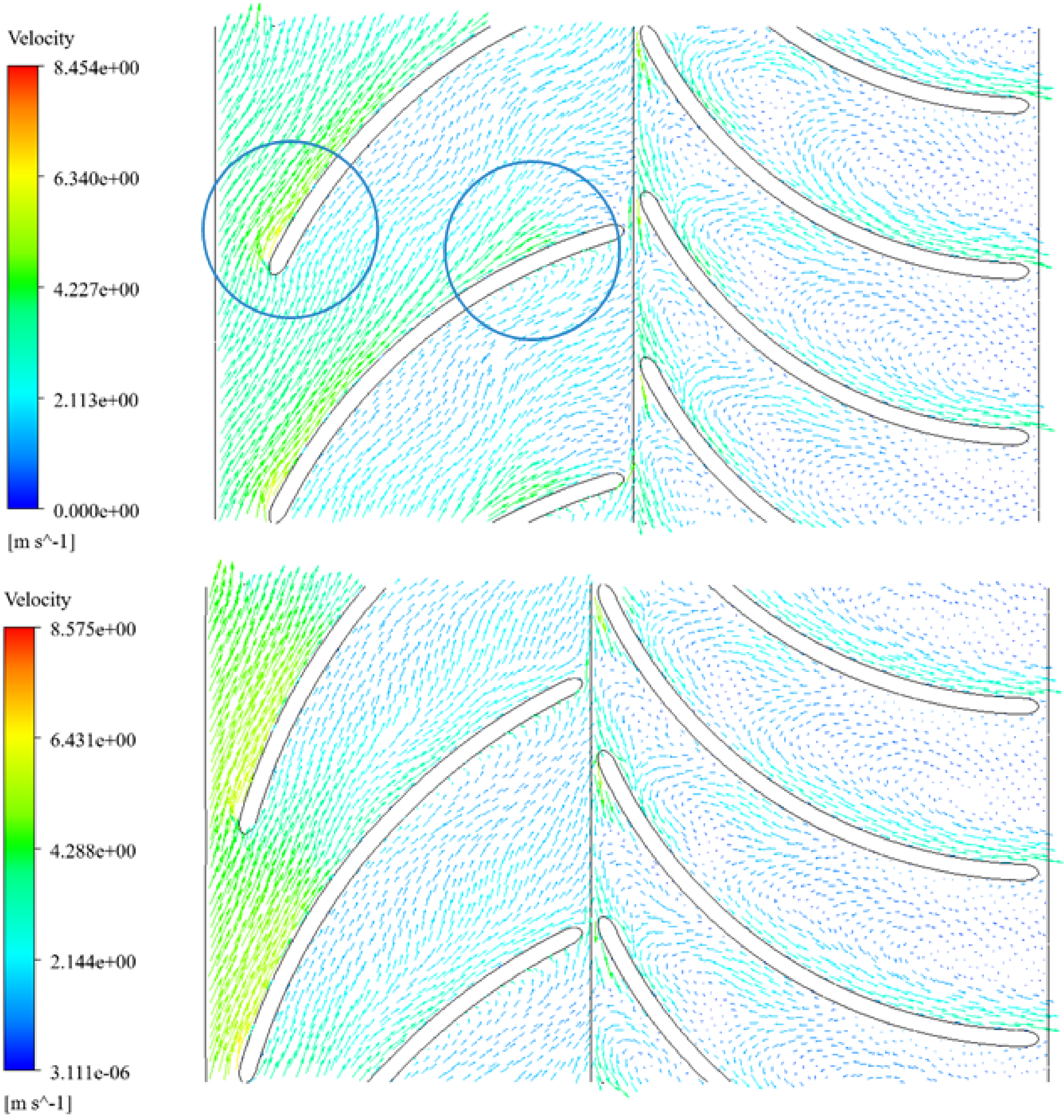

Figure 1. Flow field velocity results for (above) the previously best performing pump of the 32 base designs and (below) the newly optimised pump geometry. It is of note here that as simulations were carried out at a rotating speed of 20,000 rpm, these results do not align with the design operating point of $\Delta P = 70$ mmHg, $Q = 2L/min$, instead, these results are for $Q = 2$ L/min, $\omega = 20,000 rpm$. Highlighted by blue circle in the above image are regions of flow separation that do not occur in the impeller design for the optimised geometry shown below.
